# Transient reduction of tinnitus intensity is marked by concomitant reductions of delta band power

**DOI:** 10.1186/1741-7007-6-4

**Published:** 2008-01-16

**Authors:** Nina Kahlbrock, Nathan Weisz

**Affiliations:** 1 Department of Psychology, University of Konstanz, Konstanz, Germany; 2 Brain Dynamics and Cognition Lab, INSERM U821, Lyon, France

## Abstract

**Background:**

Tinnitus is an auditory phantom phenomenon characterized by the sensation of sounds without objectively identifiable sound sources. To date, its causes are not well understood. Previous research found altered patterns of spontaneous brain activity in chronic tinnitus sufferers compared to healthy controls, yet it is unknown whether these abnormal oscillatory patterns are causally related to the tinnitus sensation. Partial support for this notion comes from a neurofeedback approach developed by our group, in which significant reductions in tinnitus loudness could be achieved in patients who successfully normalized their patterns of spontaneous brain activity. The current work attempts to complement these studies by scrutinizing how modulations of tinnitus intensity alter ongoing oscillatory activity.

**Results:**

In the present study the relation between tinnitus sensation and spontaneous brain activity was investigated using residual inhibition (RI) to reduce tinnitus intensity and source-space projected magnetencephalographic (MEG) data to index brain activity. RI is the sustained reduction (criteria: 50% for at least 30 s) in tinnitus loudness after cessation of a tonal tinnitus masker. A pilot study (n = 38) identified 10 patients who showed RI. A significant reduction of power in the delta (1.3–4.0 Hz) frequency band was observed in temporal regions during RI (p ≤ 0.001).

**Conclusion:**

The current results suggest that changes of tinnitus intensity induced by RI are mediated by alterations in the pathological patterns of spontaneous brain activity, specifically a reduction of delta activity. Delta activity is a characteristic oscillatory activity generated by deafferented/deprived neuronal networks. This implies that RI effects might reflect the transient reestablishment of balance between excitatory and inhibitory neuronal assemblies, via reafferentation, that have been perturbed (in most tinnitus individuals) by hearing damage. As enhancements have been reported in the delta frequency band for tinnitus at rest, this result conforms to our assumption that a normalization of oscillatory properties of cortical networks is a prerequisite for attenuating the tinnitus sensation. For RI to have therapeutic significance however, this normalization would have to be stabilized.

## Background

**Figure 1:**
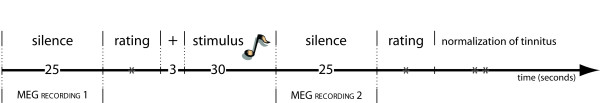
**MEG data acquisition, example trial**. This figure illustrates one of 20 trials (including 4 practice trials) every participant worked on. During the first 25 s of silence, the pre measure was obtained with MEG recording 1. A fixation cross was then followed by the presentation of one of the individually different – depending on results of the pilot study – sound stimuli (RI or CO stimulus). During the next period of silence MEG recording 2 was obtained, which was used as post measure. The mean tinnitus loudness rating (0 = tinnitus is inaudible, 10 = tinnitus is of usual loudness) refers to the preceding period of silence during which recording 2 was taken. After the second rating the participant indicated when the tinnitus reached its usual loudness again. This then started the next trial. *No time limit, fast response desired; ** time limit: 4 min.

Tinnitus – the sensation of sounds without objectively identifiable sound sources – poses a significant problem for millions of people in the world. Estimations range from 4–15% for prevalence of chronic tinnitus [1,2]. The underlying physiological mechanisms that lead to this phantom sensation are still largely unknown. From a clinical perspective, this means that no therapy reliably abolishes, or even reduces in a significant manner, the tinnitus sensation. Tinnitus seems to not be solely produced in the periphery of the auditory system, even though a hearing damage is almost always implied [3]. Studies show that after eliminating afferent input by transsection of the auditory nerve fibers, tinnitus usually persists. Even aggravations have been reported [4,5]. Eggermont and Roberts [6] pointed out that after damage to cochlear receptors, a cascade of changes is set in motion of which some could serve as a 'neural code' for tinnitus. In the central nervous system at least three correlates are discussed: enhanced spontaneous firing rates in various structures of the auditory system [7-9], altered synchronous cortical activity [10-13], and tonotopic map reorganization of the auditory cortex [11,14,15]. Regarding the latter aspect, parallel changes have been observed in the somatosensory system of phantom pain following limb amputation, leading some researchers to assume similar underlying neurophysiological mechanisms [14,16]. Nonetheless, inconsistent results were obtained for some of these correlates [8]. Furthermore, they cannot account for all aspects of the tinnitus sensation. For example, structural and morphological changes require time to develop and may then contribute to functional map reorganization in tinnitus. However, tinnitus is usually perceived immediately after an experienced noise trauma. Therefore, other explanations are needed to account for both the immediate and lasting tone perception. Synchronization of the firings of several neurons, for example, which mimics an aspect of the response to normal sound stimulation, might be promoted by decreased lateral inhibition. This could in turn enhance the synaptic efficacies through temporal integration via Hebbian mechanisms [6] leading to self-sustained tinnitus related cell-assemblies. Weisz et al [12,13] also found an altered spontaneous brain activity pattern in human chronic tinnitus subjects compared to normal hearing controls. This pathological pattern comprised a marked reduction in alpha power (8–12 Hz) and enhancement in delta (1.5–4 Hz) and gamma power (>30 Hz) at rest. These abnormalities were especially pronounced in the temporal cortical regions. Enhanced power in the slow-wave frequency range (delta frequency range; 1–4 Hz) is present in brain areas deprived of input caused by hearing damage in the case of tinnitus. This pattern of brain waves has been associated with neurological damage, for example in the perilesional areas of brain tumors and infarcts [17-19]. A reduction of this abnormal activity can be observed after the resection of such tumors [17], which predicts that in addition to the identification of dysfunctional neural networks, the mapping of abnormal slow wave activity can be used to track changes in the course of recovery or treatment [18]. This has been applied to tinnitus, and spontaneous brain activity as well. Dohrmann et al [20] manipulated the ongoing oscillatory activity (delta reduction and alpha enhancement) while leaving audiometric properties unchanged using a neurofeedback therapeutical approach. As an overall measure the tinnitus intensity decreased substantially after training. This reduced tinnitus intensity could also be noted in the follow-up sessions. Only patients who successfully modified their brain activity in both trained frequency bands, greatly reduced the intensity of their tinnitus (~70%). Thus, a normalization of abnormal rhythms in cortical networks can be associated with an alteration in tinnitus perception. The work by Dohrmann et al extends the work of Weisz et al [12] by showing that changes in the oscillatory activity can lead to perceptual changes. Yet this still leaves the question of whether the reverse also holds, i.e. whether perceptual changes also lead to changes of the ongoing oscillatory activity pattern. Tinnitus masking and residual inhibition (RI) are two tinnitus measures that can temporarily manipulate the tinnitus sensation. Most tinnitus maskers are narrow- or broadband noises. They can relieve the tinnitus sensation during their presentation, yet sometimes masking is also effective in producing a suppression or reduction of tinnitus that extends beyond the duration of the masker. This phenomenon is termed RI [21]. Masking has been used to study one patient in periods of silence from tinnitus while measuring resting magnetencephalographic (MEG) data [22]. The power spectra revealed changes in spontaneous brain activity in the auditory cortex regions in the form of a sharp theta peak in the tinnitus condition and a reduction in theta band power when the tinnitus was masked. The authors proposed that to mask the tinnitus, underlying thalamocortical circuits were depolarized, thereby reducing low frequency activity. However, the conditions are hardly comparable, as in one of them a tone was presented while in the other one no sound was presented. Additionally, oscillatory 6.5–9.5 Hz activity was found to decrease in temporal regions during sound stimulation in normal hearing individuals [23]. Therefore, masking does not seem to be an appropriate measure to study neurophysiological correlates of tinnitus intensity changes. However, during RI the period of interest is always free of an external sound stimulation. Thus, RI seems to be more adequate to study periods of reduced tinnitus intensity in chronic tinnitus sufferers, which can then be compared to other stimulation free periods. Another advantage of measuring RI is that inter-individual variability can be avoided by exploiting the possibility of comparing neural activity with and without (or at least reduced) tinnitus in one patient. A previous study has investigated neuronal processes during RI by recording spontaneous magnetic activity over the left temporal plane of one tinnitus patient. Differences in neural activity while perceiving tinnitus and during RI were thereby explored. In this study, an increase in spectral power in the low frequency range from 2–8 Hz was present during RI [24]. Recent work by Roberts et al [25] measured RI functions in relation to tinnitus spectra and auditory threshold shift. RI was assumed to be related to a desynchronization of neural activity underlying the tinnitus sensation. Maskers covering the range of hearing loss in the audiogram produced the best results in RI tests. In the only neuroimaging group study of RI published to date, Osaki et al [26] showed increases in regional cerebral blood flow in Brodmann areas 21 and 38 during RI. Both brain regions are putatively involved in auditory processing and the result could be interpreted as a transient decrease of hypoactivation or reafferentation. In the present study a period of (incomplete) RI is compared to a baseline period in which the tinnitus is of usual loudness. Changes in spontaneous brain activity are measured (see Figure 1 for an illustration of one experimental trial, see Methods for further descriptions). Based on our previous work [12,20], a decrease in amplitude in the slow-wave frequency range is expected during RI in temporal regions. An increase in amplitude in the alpha frequency range is expected in these same regions during RI compared to baseline. Changes in other brain areas and frequency bands are explored.

## Results

### Behavioral data

**Figure 2:**
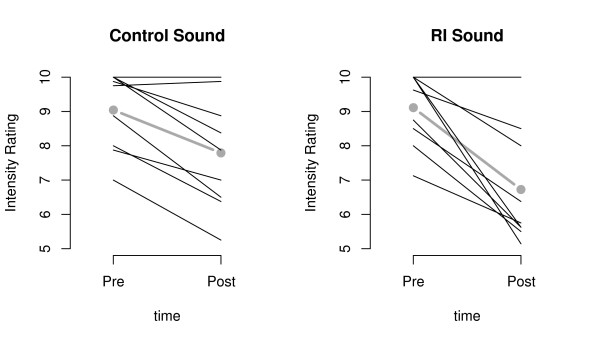
**Changes in tinnitus loudness from pre- to post-stimulation**. Mean intensity rating of tinnitus (0 = tinnitus is inaudible, 10 = tinnitus is of usual loudness) for every subject pre- and post-stimulation. One line represents one subject. The thick grey lines indicate the mean tinnitus loudness over all subjects. CO and RI conditions are shown separately. Please note that due to a decrease in tinnitus loudness over the course of the experiment, in some subjects the mean pre-tinnitus loudness is not 10 for every subject.

A highly significant main effect for time was found (F (1, 8) = 33.091, p ≤ 0.001), resulting from an overall reduction of tinnitus intensity post-stimulation, regardless of the stimulation. However, a condition (RI vs control; referred to as CO henceforth) × time (pre vs post) interaction was found to approach significance (F (1, 8) = 4.663, p = 0.063). With this interaction being in accordance with our hypotheses, post-hoc analyses were conducted. These analyses revealed that even though intensity was significantly reduced under both conditions, this reduction was slightly more pronounced for RI (RI: t (7) = 4.366, p = 0.003, CO: t (7) = 3.657, p = 0.008; see Figure 2 depicting single subject data). Please note that the mean pre-tinnitus loudness is not 10 for every subject. This limitation is due to a decrease in tinnitus loudness over the course of the experiment in some subjects).

### MEG data: spontaneous brain activity

**Figure 3:**
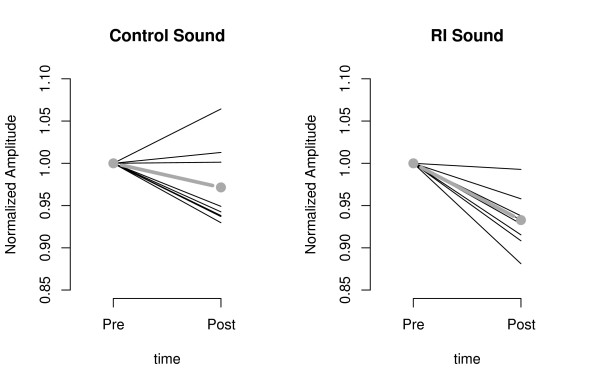
**Delta band activity at temporal sources pre- and post-stimulation**. Normalized amplitude of delta brain activity pre- and post-stimulation for CO and RI condition separately. Each line represents a single subject. The thick grey lines indicate the mean delta band activities over all subjects.

A condition × time × frequency interaction approached significance (F (3, 21) = 2.926, p = 0.058). As this interaction was in accordance with our hypotheses, further analyses were conducted. In the delta frequency band, an interaction of condition and time was found to approach significance (F (1, 7) = 3.803, p = 0.092). Additionally, a main effect for time could be observed (F (1, 7) = 25.417, p = 0.001). This leads to the assumption that the amplitude of the slow frequency range differed from pre to post but not equally for the conditions. We followed up this question by a planned comparison between pre and post separately for RI and CO conditions in the delta band in temporal regions. A highly significant (t (7) = 6.102, p ≤ 0.001) reduction of delta power was observed for RI, however not for CO (t (7) = 1.731, p = 0.127). Thus, changes in delta can be seen to be specific for the RI condition (Figure 3).

In the alpha frequency band, a main effect for time approached significance (F (1, 7) = 5.516, p = 0.051). No other effects were close to this in terms of approaching significance. As the condition × time interaction was far from being significant (F (1, 7) = 0.982, p = 0.355), the increase in amplitude can be interpreted as being non-specific. No significant effects were observed in the gamma frequency band (30.5 – 49.0 Hz and 50.3 – 70.2 Hz; F (1, 7) = 0.982, p = 0.355; F (1, 7) = 0.186, p = 0.679).

### Correlation

To test whether the observed changes in spontaneous brain activity and the reported tinnitus loudness are linearly associated a Spearman's Rho correlation coefficient for paired samples was calculated. No significant correlations between behavioral measures (reported subjective loudness of tinnitus) and spontaneous brain activity (changes in delta, contralateral temporal sources) were found (S = 116, p = 0.360).

## Discussion

To our knowledge, this is the first group study on neuromagnetic changes during RI in chronic tinnitus sufferers. Single cases, showing changes in brain activity when the tinnitus sensation was altered, have been studied during masking [22] and RI [24]. In addition, a PET study on RI revealed such changes [26]. Herein, changes in spontaneous brain activity occurring during a period of reduced tinnitus were studied. A decrease in amplitude in the slow-wave (delta) frequency range and an increase in the alpha frequency range in the temporal regions were predicted to relate to lowered tinnitus loudness.

### Behavioral data

The behavioral measures were analyzed in terms of the ability of RI and CO to induce reductions in tinnitus loudness from pre- to post-stimulus presentation. Generally, both types of stimuli induced significant tinnitus reductions. However, in accordance with the hypotheses, a strong tendency could be observed for RI to be the more potent inhibitor. One factor potentially reducing the differences of the intensity ratings between RI and CO is the instruction to focus attention on the tinnitus during periods of silence [27] (see also below).

### MEG data: spontaneous brain activity

Changes in slow-wave spontaneous brain activity in temporal regions were more pronounced in the RI than in the CO condition. Slow-wave spontaneous brain activity decreased from pre- to post-stimulation in the RI, but not in the CO condition. In the alpha frequency band, such a condition specific effect could not be found. These findings are partly in accordance with previous works by our group. In a more general sense it is also consistent with findings by de Jongh et al [17], who reported a reduction of initially enhanced delta activity in the region around a treated tumor. An amelioration of pathological conditions can thus lead to a normalization of abnormal rhythms present in cortical networks affected by the lesion. Contrary to expectation the amplitude in the alpha frequency band was not augmented in a state of reduced tinnitus loudness (RI). Also, gamma activity did not show significant changes despite our previous finding implying an important role of gamma in the emergence of the phantom sound [13]. One possible explanation for this is that the gamma frequency ranges were defined too broadly and that effects could be specific to even more circumscribed frequency ranges. Furthermore, this frequency range may differ inter-individually to some extent eliminating the identification of systematic effects on a group level. To tackle this important issue a within-subject analysis would be necessary. However, due to the low amount of trials per condition the current design is not appropriate this kind of analysis. Future studies shortening the masker and post-masking period, concentrating fully on the early (< 5 s) post-masker period will shed more light on this question. However, it is also plausible that – after settling methodological issues (e.g. verbal reports of intensity versus 'objective' audiometry) – tinnitus intensity changes are not reflected in local gamma activity but rather depend on the synchronization of a distributed neuronal network similarly to what we have recently shown for tinnitus intrusiveness [28]. Weisz et al [29] have recently proposed an *Oscillatory Model of Tinnitus *(now termed synchronization by inhibition modulation; SIM), in which a conceptual link was established between the three frequency bands (delta, alpha, and gamma) in the context of tinnitus. In this framework, delta activity reflects the general deprivation mediated slowing of activity, whereas gamma reflects the synchronized firing of neurons necessary for the emergence of a conscious sensation. The latter is due to a reduction of inhibitory neuronal activity that is putatively reflected as alpha rhythm. The present study shows that changes in the slow-wave frequency band do not necessarily induce simultaneous and measurable changes in other bands. A plausible reason for this is that the fundamental oscillatory processes that are associated to the perception, i.e. the enhanced gamma, as well as the reduced alpha activity, become self-sustained if the pathological condition persists. A transient reafferentation, as indicated by the delta reduction, will then not suffice to fully break up the maladaptive oscillatory pattern. This finding confirms anecdotal impressions from clinical practice, that hearing aids may reduce the intensity of tinnitus. However, hearing aids – even if used for a long time – are not known to completely eliminate the tinnitus sensation. Another explanation why simultaneous changes were observed only in the delta band could be (as mentioned previously) inherent to the design of the study itself. In order to obtain ratings of intensity, participants were always required to focus their attention on the tinnitus sensation. It is likely that attention was even enhanced post-stimulation, because the participants had to note changes of intensity. This could have induced oscillatory brain activity that counteracts that induced by the stimuli alone. Indeed reductions of alpha power as well as gamma enhancements have been frequently reported during tasks of focused attention [30,31]. This fits well with the observation in our present study that alpha activity was reduced post-stimulus, however not differently for the conditions. In future studies a passive RI condition should be examined in order to minimize attention effects and thus, unspecific changes in alpha. RI functions would need to be tested in advance and an estimation of tinnitus loudness during the experiment should be given after the last trial. The assumption that tinnitus loudness and spontaneous brain activity are linearly related was investigated. The observed changes in spontaneous brain activity and the reported tinnitus loudness were not correlated (S = 116, p = 0.360). The strength of the reduction in delta amplitude at the contralateral temporal sources after stimulus presentation did not consistently predict the strength of change in the tinnitus sensation. This non-significant correlation could be easily explained by the only marginal behavioral differences between RI and CO conditions. It could also be due to the very small number (n = 8) of subjects.

### Limitations

It cannot be excluded that subjects were distracted to some extent while concentrating on their tinnitus. Also, placebo effects elicited by the stimuli are difficult to rule out experimentally because with audible presentation of the masking sounds participants are not blind to the type of stimulus and may form specific expectations tied to the type of sound. Although RI was induced in the subjects, it might have been rendered deeper and longer-lasting with the use of higher volumes and frequencies and longer durations of stimulation. An even clearer behavioral contrast between RI and CO would be preferable to more thoroughly study RI effects on neural processes. Furthermore, it is a limiting factor that for some subjects the tinnitus sensation decreased in the course of the experiment, therefore probably reducing the potency of our RI stimuli. It should be emphasized that in this work we defined RI as partial tinnitus reduction. Based on our pilot study (data available on request), we were not able to identify subjects that showed a complete abolishment of the tinnitus sensation for a significant time period. Herein, the question of whether RI is a "period without/of reduced tinnitus" or is qualitatively different from not having/having reduced tinnitus, remains unanswered. As is the question of whether changes in the power spectrum actually represent the neurophysiological correlate of tinnitus, or rather develop in response to perceived changes in tinnitus loudness [24]. The observed changes in the power spectrum could be a reaction to changes in auditory perception as auditory stimulation triggers changes in auditory cortex responses [32]. If tinnitus is viewed as permanent auditory stimulation that is withdrawn for a period during RI, one could come to this conclusion. Thus, it seems necessary to investigate whether RI really leads to a transient state of normalization of brain functioning. It is known that tinnitus sufferers have an abnormal pattern of spontaneous brain activity [12]. The present study showed that this pathology changes in response to a reduction in tinnitus loudness. Thus, it seems reasonable to assume that these changes do represent the neurophysiological correlate of tinnitus. A masking study [22] corroborates this conclusion by showing that the power spectrum of a tinnitus sufferer changed during a reduction in tinnitus loudness with auditory stimulation being present. Finally it has to be stated that very extended and deep RI is an overall rare phenomenon (~25% with our relatively soft criteria), thus raising the question of the representativity of the sample for the entire tinnitus population. That said, the prospect of potentially gaining valuable generalizable insights regarding comparable periods of normal and reduced tinnitus makes the study of RI a worthwhile enterprise. It will however have to be backed up in future by studies focussing on short periods following masker presentation in tinnitus participants with little or virtually no RI.

## Conclusion

The aim of this study – to analyze neural activity during reduced tinnitus loudness without sensory stimulation confounding these measures – was achieved. The determination of whether the observed changes in spontaneous brain activity stem from the reduction of the tinnitus sensation or the sound presentation is thereby simplified. Despite limitations, the results of this study suggest that a reduction of the tinnitus perception leads to changes in the oscillatory properties of cortical networks connected to tinnitus. In particular, changes in slow-wave frequencies appear to be RI related. Large-scale synchronous neuronal activity as reflected in ongoing spontaneous brain activity could relate to the immediate and lasting tone perception of tinnitus. Its instantaneous changes when altering the tinnitus perception as well as the finding of Dohrmann et al [20], imply a bidirectional influence. This has important clinical implications, indicating that successful causal treatment approaches would need to permanently interrupt the underlying oscillatory pattern. At this point it is unclear whether and how effects of input based ('bottom-up') approaches such as RI could be extended. One possibility would be to try to combine such approaches with input-independent ('top-down') approaches such as neurofeedback [20], which could potentially uphold effects even during periods without input.

## Methods

### Participants and pilot study

A pilot study (n = 38, 14 female, 24 male, mean age 53.71, SD: ± 10.36) was conducted in order to find chronic tinnitus sufferers showing RI. To quantify the individual tinnitus related distress, all subjects completed the 'Tinnitus Fragebogen' (TF; [33]; adapted from the Tinnitus Questionnaire [34]). An extensive audiometric testing was conducted determining the amount of hearing loss, a diagnosis of dead regions, and a tinnitus spectrum [35]. Masking levels were tested for the following frequencies: 0.84, 1.201, 1.715, 2.45, 3.5 and 5.0 kHz. The masking stimuli (at individual masking levels) were then used as stimuli to test RI functions. Of the 38 subjects in the pilot study, 10 (4 females and 6 males) fitted the criteria for the actual study (see below for derivation of criteria). Nine of them took part in the actual study (Table 1). All participants were right-handed according to the Edinburgh Handedness Inventory [36]. Subject 2 fell asleep during the MEG recordings. His data were therefore not considered for further analyses. The statistical analyses are therefore based on the data from eight subjects (four female, four male, mean age: 53, SD: ± 12.01). Further details of the pilot work are available from the authors on request.

**Table 1:** Subjects participating in the study. Characterization of the subjects who experienced a reduction of their tinnitus of at least 50% lasting a minimum of 30 s after masker offset in the pilot study, who then took part in the actual study. Subject 2 was excluded from data analysis, as he fell asleep during data collection

**Subject**	**Age**	**Sex**	**Etiology**	**Tinnitus dominance**	TF* score	**Tinnitus duration (years)**	**RI frequency (Hz)**	**CO frequency (Hz)**
1	57	F	Cold	Left	43	1	5000	3500
2	49	M	Stress	Bilateral	23	0.5	2450	840
3	29	M	Hearing loss	Right	22	4	840	5000
4	62	M	Unknown	Bilateral	11	8	2450	840
5	47	M	Unknown	Left	15	7.5	3500	840
6	65	M	Unknown	Left	12	1.5	2450	5000
7	47	F	Stress	Bilateral	14	12	1715	840
8	64	F	Unknown	Left	21	5	1715	5000
9	53	F	Hearing loss	Right	63	2	2450	5000

*TF score (distress): 0–30: minor; 31–46: moderate; 47–59: severe; 60–84: very severe.

In recently published work [25] a mean duration of 32.2 s of partial RI was found for the best RI eliciting stimulus. A reduction in tinnitus loudness of more than 50% for at least 30 s after the stimulus is referred to as RI herein. One of the frequencies not eliciting RI was used as CO stimulus. If more than one stimulus fitted the criteria of the CO stimulus the frequency furthest away from the RI frequency was used.

Prior to the experiment all subjects gave written informed consent to participate in the MEG study. They were informed that they could discontinue the experiment at any time (e.g. when sounds were too discomforting) without the emergence of any disadvantages for them (e.g. regarding payment or prospect to participate in future treatment studies). The procedures conformed to the ethical principals set out in the Declaration of Helsinki and were approved by the Internal Review Board of the University of Konstanz.

### Stimuli and preparation of subjects

To monitor the subjects' head positions relative to the sensor and potential head movements, five coils were attached to their heads. Individual head shapes were digitized using a 3D position tracker (3 Space^® ^Fastrack^® ^Polhemus, Colchester, VT, USA). To control for horizontal and vertical eye movements an electrooculogram (EOG) was recorded using four electrodes attached to the left and right outer canthi and above and below the right eye. Two electrodes recorded an electrocardiogram (ECG) in order to correct for heart beat artifacts in the MEG signal. For auditory stimulation two plastic tubes were used. Prior to the actual experiment the participants adjusted the intensity of the RI sound until it could mask their tinnitus. To control for loudness differences among conditions, the CO stimuli were adjusted so that their loudness was equal to the perceived loudness of the experimental stimuli. The sound stimuli used in this study had a relative bandwidth that changed according to the frequency (0.2 × center frequency). The patients were stimulated binaurally. Consistent with earlier reports [25], the sound stimuli were presented for 30 s.

### Experimental setup

The participants lay still in the MEG chamber with dimmed lighting during data collection. A video beamer (D-ILA, DLA-G11, JVC, Friedberg (Hessen), Germany) and a system of mirrors were used to project the instructions to the ceiling of the chamber. The auditory stimuli were presented analogous to the stimuli in the masking experiment. The first part of the experiment consisted of a 25 s period of silence. The participants lay still and listened to their tinnitus. After this resting period, the participants were asked to indicate the mean tinnitus loudness during the resting period on a scale ranging from 0 to 10 (0 = tinnitus is inaudible, 10 = tinnitus is of usual loudness). After the rating, a fixation cross appeared for a duration of 3 s, which was then followed by the presentation of either the RI or the CO stimulus (30 s). The RI and the CO tone were each presented 10 times in a pseudorandom order. The stimulus presentation was followed by another resting period (25 s) and rating. After this rating the subjects indicated when the tinnitus reached its usual loudness again and a new trial was started. Each subject worked through 20 trials, with the first 4 being practice trials that were not included in the analyses. For the visualization of one trial see Figure 1. The behavioral protocol was implemented in PsyScope X [37].

### MEG recording

Using a 148-channel neuromagnetometer (Magnes 2500 WH, Biomagnetic Technologies, San Diego, CA, USA) 25 s pre- and 25 s post-stimulus of resting MEG (sampling rate: 678.17 Hz; 0.1–200 Hz band-pass filter) were recorded in every trial. The participants were requested to keep their eyes open and to maintain gaze on a fixation mark on the ceiling of the recording chamber. With a video camera installed in the chamber the subjects' behavior during data collection was monitored. A Synamps amplifier (Neuroscan, Sterling, VA, USA) was used to record the EOG and ECG.

### Data processing

The MEG data were noise reduced by removing magnetic noise generated from non-biological, external sources, registered by MEG reference channels from the raw data. With a multiple source approach [38] implemented in BESA^® ^(Brain Electric Source Analysis, MEGIS, Graefelfing, Germany) blink and heart beat artifacts were removed from the MEG signal. Noisy channels were interpolated in BESA^® ^using spherical spline interpolation. The surface MEG was transformed into brain source activity, using a source montage [28] in the source analysis module of BESA^®^. The montage consisted of eight regional sources distributed over the brain in a fairly even manner to represent compound activity from all major brain regions: two in the left and right temporal planes, two in the left and right prefrontal areas, two in the left and right parietal lobes, one in the middle posterior region, and one medially approximately between the parietal and the prefrontal sources. The montage was used for all subjects and was adjusted to the individuals' head sizes. The source data were exported to MATLAB^® ^(version 7.0.4.365, The Mathworks Inc., Natick, MA) for further analyses. Amplitudes were calculated for each of the eight sources with a mean fast Fourier transformation (FFT). The FFT as a signal processing method decomposes time-varying signals to frequency space from which amplitude and phase information can be extracted. Before applying the FFT, 1000 ms was subtracted from each block's start to avoid an offset response (512 data points; i.e. approximately 755 ms; with a 50% overlap). After calculation of amplitudes of the spontaneous brain activity, frequency bands were defined as delta (1.3–4.0 Hz), alpha (8.0–12.0 Hz), low gamma (30.5–49.0 Hz), and high gamma (50.3–70.20 Hz). Normalization of the data was done by calculating mean amplitudes for pre- and post-test values for every subject over the whole power spectrum (every source and condition separately). The amplitudes of the power spectra were then divided by these mean amplitudes. For every subject mean values were calculated for the whole recording period. Thus, for example, one value was given for the post-stimulus delta condition averaged over the whole 24 s and all trials of one condition (RI vs CO) of analyzed data for every subject. The latter-described comparisons were between these average pre and post values at the temporal sources contralateral to the ear with the dominant tinnitus sensation for subjects with one-sided tinnitus, and the average of values at left and right temporal sources for the two subjects with bilateral tinnitus.

### Statistical analysis

Statistical analyses were conducted using the statistical software R [39]. For the behavioral data a two-way analysis of variance (ANOVA) was calculated with factors time (pre- vs post-stimulus period) and condition (RI vs CO). The dependant variable was the change in tinnitus loudness. Post-hoc two sided paired t-tests were conducted. For the MEG data, mean power spectra (for the pre and post period), averaged over all trials, were calculated for every person. The amplitude of spontaneous brain activity at the temporal sources contralateral to the ear with the dominant tinnitus perception was chosen. In the case of bilaterally equally intensive tinnitus the mean of left and right temporal sources was calculated. A three-way ANOVA was calculated with factors time (pre vs post), condition (RI vs CO) and frequency (delta vs alpha vs low gamma vs high gamma). Post-hoc two sided paired t-tests were calculated for the differences in amplitude between pre- and post-stimulation in the RI and the CO condition in delta. A Spearman's Rho correlation coefficient for paired samples was calculated to further clarify the connection between behavioral and physiological data.

## Authors' contributions

NK participated in the design of the study and in implementing some of the scripts for data collection, ran the data collection, performed the statistical analysis and interpretation of the data, drafted the manuscript and participated in revising it critically. NW conceived of the study, designed it, participated in its coordination, implemented most of the scripts for data acquisition, participated and helped in the analysis and interpretation of data, and revised the manuscript critically. Both authors read and approved the final version of the manuscript.
